# Reporting methods in studies developing prognostic models in cancer: a review

**DOI:** 10.1186/1741-7015-8-20

**Published:** 2010-03-30

**Authors:** Susan Mallett, Patrick Royston, Susan Dutton, Rachel Waters, Douglas G Altman

**Affiliations:** 1 Centre for Statistics in Medicine, University of Oxford, Linton Rd OX2 6UD, Oxford, UK; 2 MRC Clinical Trials Unit, 222 Euston Road, London NW1 2DA, UK

## Abstract

**Background:**

Development of prognostic models enables identification of variables that are influential in predicting patient outcome and the use of these multiple risk factors in a systematic, reproducible way according to evidence based methods. The reliability of models depends on informed use of statistical methods, in combination with prior knowledge of disease. We reviewed published articles to assess reporting and methods used to develop new prognostic models in cancer.

**Methods:**

We developed a systematic search string and identified articles from PubMed. Forty-seven articles were included that satisfied the following inclusion criteria: published in 2005; aiming to predict patient outcome; presenting new prognostic models in cancer with outcome time to an event and including a combination of at least two separate variables; and analysing data using multivariable analysis suitable for time to event data.

**Results:**

In 47 studies, prospective cohort or randomised controlled trial data were used for model development in only 33% (15) of studies. In 30% (14) of the studies insufficient data were available, having fewer than 10 events per variable (EPV) used in model development. EPV could not be calculated in a further 40% (19) of the studies. The coding of candidate variables was only reported in 68% (32) of the studies. Although use of continuous variables was reported in all studies, only one article reported using recommended methods of retaining all these variables as continuous without categorisation. Statistical methods for selection of variables in the multivariate modelling were often flawed. A method that is not recommended, namely, using statistical significance in univariate analysis as a pre-screening test to select variables for inclusion in the multivariate model, was applied in 48% (21) of the studies.

**Conclusions:**

We found that published prognostic models are often characterised by both use of inappropriate methods for development of multivariable models and poor reporting. In addition, models are limited by the lack of studies based on prospective data of sufficient sample size to avoid overfitting. The use of poor methods compromises the reliability of prognostic models developed to provide objective probability estimates to complement clinical intuition of the physician and guidelines.

## Background

Prognosis is central to medicine. Clinicians use patient and disease characteristics to inform patient treatment and predict patient outcome. Development of prognostic models enables identification of variables that are influential in predicting patient outcome and the use of these multiple risk factors in a systematic, reproducible way according to evidence based methods [1].

The goal of a prognostic model is to provide quantitative knowledge about the probability of outcomes in a defined patient population for patients with different characteristics [2]. With a multivariable model, predicting a patient's future outcome can be made using combinations of multiple patient risk factors.

Models are ideally developed based on a combination of prior knowledge of the disease with judicious and informed use of statistical methods. Many of the multiple steps involved to develop a prognostic model can lead to flawed or biased models if used without good statistical understanding.

This article examines the methods used in developing prognostic models by a systematic review of 47 published articles including prognostic models where the aim of the article was to develop a new prognostic model as a combination of two or more independent risk factors to predict patient outcome. We focussed on study design, definition of outcomes, coding of variables and statistical methods used to develop the model. We have set out findings in the context of the methodological literature in which the impact of the different methods on model predictions has been studied. Although there are no specific guidelines on developing prognostic models, there are some excellent books and articles providing advice on good and poor methodology [1-5]. However, our study shows widespread use of poor methods in current studies. We aim to highlight this and to add to the methodological literature to inform and prompt further improvements in model building.

## Methods

### Literature search

We had planned a hand-search of 10 high impact cancer journals in 2005 for our sample of articles (*Journal of the National Cancer Institute, Journal of Clinical Oncology, International Journal of Cancer, British Journal of Cancer, Cancer, European Journal of Cancer, Annals of Oncology, Clinical Cancer Research, Cancer Research, International Journal of Radiation Oncology*). These were the higher impact oncology journals identified as publishing a reasonable number of prognostic studies (2005 impact factors range 3.7 to 15.2). However, there were only 11 articles that met our inclusion criteria, and only two journals (*Journal of Clinical Oncology *and *Cancer*) with more than one article in 2005. As we were unable to identify our target of approximately 50 articles in these journals, we decided to design a search string in order to search all journals.

We used the prognostic articles identified from hand-searching the *Journal of Clinical Oncology *to design our search string (Additional file 1). We evaluated the search string on the 2005 issues of *Cancer*, comparing the number of articles found by string search and hand-search.

A hand-search of the titles and three line journal summaries of 784 articles in *Cancer *identified 42 articles as potentially eligible. On reading the abstracts, 16 full papers were read to establish eligibility, and four articles were found to meet inclusion criteria. Using our search string, 74 titles and abstracts were identified, 12 full papers read and 5 articles identified that met inclusion criteria. Use of the search string identified relevant articles with considerably less work than the hand-search. As all articles in *Cancer *from the hand-search were identified with the electronic search, as well as an additional article, the search string had adequate performance for this study, as we wanted a good representation of relevant articles rather than all possible articles. The search string was then used to search PubMed for articles published in 2005. There was no language restriction in our search. Our search string is reported in Additional file 1.

### Inclusion criteria

We included articles that met our inclusion criteria: development of a prognostic model in cancer patients; where the outcome was the time to an event; where the aim of the research was to develop a new prognostic model as a combination of at least two variables to predict patient outcome; and data were analysed using multivariable analysis suitable for time to event data. Articles that included only validation of a pre-existing model, were not published in print in 2005, or were based on microarray, gene profiling or proteomics methods were excluded. Queries on article inclusions were referred to second readers (PR, DA).

### Validity assessment and data extraction

We aimed to assess a sample of approximately 50 articles developing prognostic models in cancer, to provide a good descriptive review of the current literature including a range of cancer sites, authors and journals. We judged little further value would be obtained from a larger sample. We assessed articles in random order using a piloted data extraction form based on key aspects of model design and development from the current literature [1,3,4,6,7].

Data items extracted for this paper included: study design, sample size, number of patients and events, outcome definition, number and coding of variables in model, and methods of selection of variables. In a companion paper, Mallett et al [8], we report the assessment of methods and reporting of multivariable analysis, numerical and graphical presentation of the model, creation of prognostic index and risk groups, model discrimination and calibration, methods of validation, and usability of reported model. Data extraction forms are available from the corresponding author.

Sixteen items were extracted by duplicate data extraction by two of three reviewers (SM, SD, RW) with reference to a third reader where necessary. One reviewer (SM) assessed all articles and all items. For the three items on methods of variable selection, data were extracted by one reader (SM). If more than one model was presented in an article, the first model reported in title, abstract or text was selected.

## Results

### Literature search

Articles on prognostic models are hard to identify in electronic bibliographic databases as there is no standard medical subject heading from the U.S. National Library of Medicine's controlled vocabulary (MeSH heading) term comparable to that identifying randomised controlled trials (RCT) and no standard nomenclature. Several search strings have been developed [9-11] but other studies have used hand-searching [12].

**Figure 1:**
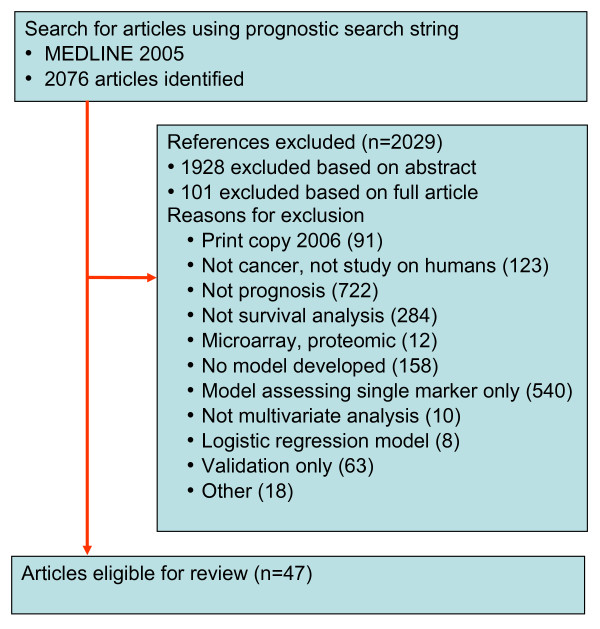
**Flowchart of articles**.

Our search string selected 2,076 articles from 681,530 articles with subject MeSH heading of *neoplasm *published in 2005, of which 47 met inclusion criteria for this study (Figure 1) [13-59]. The yield of articles identified in the search string that met our study inclusion criteria was 2.3%, higher than alternative search strings. The performance of other prognostic search strings to identify relevant articles is shown in Additional file 2.

In the 47 articles included in our study, prognostic models were developed for the following cancer sites: urological (ten articles); breast (seven); haematological malignancies (six); upper gastrointestinal and pancreas (five); prostate (five); bone metastasis (five); lung (three); head and neck (two); colorectal (one); skin (one); ovary (one); and bone and soft tissue (one).

### Data extraction

Across 16 data extraction items relating to reporting of model performance measures, there was agreement between readers for 82% of items. Thirty-eight percent of the differences were caused by ambiguities in the articles, often due to difficulties in extracting from the articles the numbers of variables, patients or events used in the model.

### Study design

The best study design to use for development of a prognostic model is a prospective cohort study [2], where baseline variables can be measured for all patients and selection bias due to incomplete availability of data or archival samples can be avoided [60]. Randomised controlled trial study designs can be appropriate if eligibility criteria are not too restrictive so as to lessen transportability, and if the sample size assessed by the number of events is adequate. For randomised controlled trials where there is a treatment effect, it is recommended that the treatment arm is included among the predictor variables [2].

In 21% (10) of articles models were based on prospective cohorts [16,21,22,26,34,43,45,46,56,59] (Table 1), two of which were based on phase II trials [16,45]. Six of these studies reported the same number of eligible and included patients. Patients recruited from RCTs in 11% (5) of the studies [14,38,40,44,47]. In three RCTs researchers reported there was no significant treatment effect [14,40,44] and in one study it was explicitly stated that treatment arm was not used as a variable for this reason [40]. Sixty-eight percent (32) of the studies used a retrospective cohort design from clinical records. Ten of these studies had the same number of eligible patients as included patients, suggesting that complete patient data were an unspecified inclusion criterion [24,27,30-32,36,38,48,51,55]. In one article authors analysed three sets of data separately, two from RCTs and one from retrospective database records [38].

**Table 1:** Study design and size

	% (n = 47) articles
Study design	
RCT*	11 (5)
Prospective cohort study	21 (10)
Database or other retrospective cohort	68 (32)

Reason for sample size	
No reason given	77 (36)
Justified time interval (clinical or technology)	4 (2)
Trial size (RCT or cohort)	19 (9)

Power calculation reported	0 (0)

	**Median (IQR) [range]**

Eligible for study:	
Number of patients (n = 35)	403 (148 to 833)
Number of events (n = 20)	112 (62 to 289)
Included in analysis:	
Number of patients (n = 45)	342 (130 to 684)
Number of events (n = 33)	110 (61 to 230)

Number of events per candidate variable (n = 28)	10 (5 to 33) [2 to 95]

*RCT randomised controlled trial

### Number of patients meeting inclusion criteria

The reporting of the number of patients meeting inclusion criteria, as opposed to those selected for inclusion in the analysis, can be helpful in assessing the potential for selection bias in retrospective studies. The representativeness of the patient sample cannot be judged if data availability is used as an eligibility criterion [12].

In 36% (17) of the studies, authors reported a difference in the number of patients meeting inclusion criteria from those included in the study. In 36% (17) of the studies the number of patients was the same. This was highly suggestive that inclusion criteria included availability of all prognostic variables, except perhaps for two studies where RCT study data with no exclusions had been used [25,40]. In 28% (14) of the studies the number of patients or events that were eligible for the study was either not reported or was unclear.

### Number of patients and events included in analysis

The number of patients and the number of outcome events in the patient population used to develop a prognostic model are key study details that it is critical to report. Although the number of patients recruited to a study is always important to know, the statistical strength of a prognostic study is driven by the number of events and by the prognostic ability of the individual predictors.

The number of patients included in the analysis was clearly reported in 96% (45) of the studies. Of the other two studies, it was reported in one study that the patient population had been split into development and validation datasets but how many were in each dataset was not reported [13,26].

The median number of patients included in analysis was 342 (IQR 130 to 684, n = 45) for model development. The number of events included in the analysis was not reported in 30% (14) of the studies. In the studies where it was reported, the median number of outcome events was 110 (IQR 61 to 230, n = 33).

### Sample size and events per variable

A commonly used *rule of thumb *to judge sample sizes for developing prognostic models is the recommendation of at least 10 events per variable. This rule is based on estimates of the stability of coefficient estimates for individual variables in prognostic model [61-66]. Many prognostic studies have unsuitably small sample sizes, identified easily by the rule of thumb as having fewer than 10 events per variable used in model development.

Use of variable selection procedures in studies with small sample sizes results in models with biased selection of variables, unreliable coefficients and inaccurate predictions [3]. Despite the difficulties in calculating suitable sample sizes with power calculations, studies should report their justification for the choice of sample size.

In 77% (36) of the studies no justification was given for the sample size. In 23% (11) of the studies reasons were given for sample size; with one exception all these studies were RCTs or prospective cohorts [14,16,25,26,38,40,43-45,54,56]. In two studies researchers used a justified time interval based on a change in treatment or chemotherapy regimen [43,54]. Two trials used a subset of disease categories, one from an RCT and one from a time justified database [25,54]. None of the studies included a power calculation.

For the 28 studies (60% of studies) where it could be calculated, the median number of events per candidate model variable (EPV) was 10 (IQR 5 to 33, range 2 to 95). In 30% (14) of studies too few events were reported for the number of variables analysed in the model, indicating insufficient data for reliable interpretation of model findings. In 40% (19) of the studies there was insufficient information to calculate study EPV.

### Definition and number of outcomes

Clear definition of the study outcomes in prognostic research is critical for a clear interpretation of the model. Studies in breast cancer [67] and treatment of bone metastasis [68] have found that outcome definitions are often unclear and inconsistent between studies. Disease free survival (DFS) is particularly inconsistent and can include any or all of local, regional, distant recurrence and death [67].

The use of study outcomes defined *a priori *is the recommended standard in order to avoid any suggestion of selective reporting of model outcomes based on study results. Articles where a large number of study outcomes are examined have potential for bias in the selection of outcomes for multivariable analysis and the prognostic index. A review of randomized controlled trials identified selective outcome reporting where the prespecified primary outcome of event -free survival was omitted from published reports [69].

Overall survival was examined as the primary outcome in 66% (31) of the studies (Table 2). DFS (or the related outcomes of event-free survival local regional recurrence etc.) were examined in 34% (16) of the studies. A clear definition of the outcomes examined in the multivariable analysis was included in 60% (28) of studies. In 40% of the articles the outcomes were not clearly defined; in 30% (14) of studies it was unclear whether all deaths or only cancer deaths were included in overall survival and in 10% (5) of the studies whether death was included in DFS outcomes.

**Table 2:** Outcomes and definitions

	% (n = 47) articles
Primary outcome	
Overall survival	66 (31)
Disease free survival (DFS)	34 (16)

Definition of outcomes	
Overall survival (n = 31)	
Explicitly death from any cause	15 (7)
Cancer death only	21 (10)
Type of death unclear	30 (14)
Disease free survival (n = 16)	
DFS including death*	10 (5)
DFS not including death	13 (6)
DFS but unclear if includes death	10 (5)
Multivariable outcome clearly defined‡	60 (28)

* In one study it was not clear if DFS deaths included all deaths or only cancer specific deaths

‡ In two articles it was unclear how many outcomes were examined

In our study there was a median of one outcome per article (IQR one to two; range one to five; Table 2). In 13 studies researchers reported more than one outcome, with three or more outcomes examined in four studies. The median number of outcomes included in multivariable analyses was one (IQR one to one; range one to five). In 13 studies researchers used more than one outcome in multivariable analyses, in three studies researchers used three or more outcomes in multivariable analyses. In two studies it was unclear how many outcomes were used in multivariable analyses [32,33], but two and three outcomes were included in these papers.

### Coding of variables

Multivariable analysis may be performed using a mixture of continuous, categorical and binary candidate variables. The methods used to handle the continuous and categorical variables may strongly affect the final prognostic model, both in terms of variables selected by their statistical significance and the coefficient values for included variables [5].

The practice of dichotomising continuous variables is not recommended, as it is an extreme form of rounding that causes loss of information and statistical power, equivalent to losing a third of the data [70]. In addition it results in unrealistic steps in the predicted risk, with patients at either side of a cutpoint categorised with very different levels of risk [70]. Dichotomising at the median value is problematic as each study will have a different cutpoint and cutpoints are data driven, that is, chosen using the data itself [71]. Furthermore choice of an optimal or minimal p-value cutpoint is even worse [72]. Despite these and other disadvantages, dichotomisation of continuous variables is very frequently used in developing prognostic models. Classification of continuous variables into three or more groups as ordinal variables will similarly affect variables selected by significance in a prognostic model.

Continuous candidate variables were frequently converted to categorical or binary variables. All studies included at least one continuous candidate variable in model development. Only one article kept all continuous variables as continuous (Table 3), whereas 19% (9) categorised at least one continuous variable, 51% (24) categorised all continuous variables and in 28% (13) of the studies it was unclear how continuous variables were handled due to poor reporting.

**Table 3:** Variables in multivariable analysis

	% (n = 47) articles
Coding of variables in model	
Coding explicit for all candidate variables	68 (32)
Coding explicit for all variables in final model‡	70 (33)

Coding of continuous candidate variables	
All continuous	2 (1)
All categorised/dichotomised	51 (24)
Combination of continuous and categorised variables	19 (9)
No continuous candidate variables	0 (0)
Unclear/not reported	28 (13)

	**Median (IQR)** **(range)**

Number of variables	
Candidates used to develop model* (n = 46)	11 (7 to 14) (2 to 27)
Included in final model** (n = 44)	4 (3 to 6) (2 to 12)

‡ Two papers could not be assessed as the final model was unclear.

* In four articles variables were excluded where it was unclear if these were candidate variables. In one article the number of candidate variables was completely unclear

** Three articles could not be included: two because the final model was not presented in the paper, one because it was not clear which of the alternative models in the paper was the final model.

Clear and transparent reporting of how variables are coded in the model is necessary so the model can be repeated and applied in clinical practice [73]. The coding of all the candidate variables used in model development was reported in 68% (32) of the studies (Table 3) and the coding for the variables in the final model was reported in 70% (33) of the studies.

### Selection of variables in multivariable analysis

A description of patient demographics, disease severity, laboratory investigations and treatment is important in understanding the clinical population used to develop a prognostic model. Many of these characteristics are used as variables in development of the prognostic model, with statistical modelling used to select the most influential variables for a final model.

There is no consensus on the best methods for selecting variables for a final model, although some methods are particularly cautioned against, such as inclusion or exclusion of variables based on univariable analysis [4]. Selection conditional on univariable tests in small sample sizes is likely to introduce error as the correlation between prognostic variables is not properly controlled for [74]. An additional, but lesser, concern is that prognostic variables are eliminated which have a weak association with study outcome or are by chance not prognostic in the particular sample of patients.

It is recommended that prognostic risk factors which have clinical credibility and are already well established in the literature are retained in models. Biases are generated by the method of selection and the p-values used to set selection limits for inclusion and exclusion of variables [3-5]. If automated variable selection methods are to be used, then backward elimination is preferred to forward selection as it starts with a full model and considers a wider range of possible best models, and also is a better method where variables are correlated [5].

We assessed the methods used for selection of variables in the 43 studies using Cox models for development of a prognostic index (Table 4). Twenty-six percent (11) of Cox models included all variables in the multivariable analysis [13,28,33,35-37,39,40,46,50,56]. Fifteen percent (6) of the studies used non statistical reasons, including information from published literature and investigators' choice to select variables for multivariable analysis [16,23,26,27,38,44]. Specific reasons to exclude variables were based on collinearity with other variables, due to missing data or due to variables relating to treatment where the model was to predict regardless of treatment. In 49% (21) of the studies, researchers used a method that is not recommended, which consists of a conditional pre-test for statistical significance in univariable analysis. In 11% (5) of the models, the method of variable selection was not reported or was unclear.

**Table 4:** Selection of variables in multivariable analysis

	% (n = 43*)
**Selection of variables for inclusion in multivariable analysis**	
All candidate variables used (no selection)	26 (11)
All candidate variables apart from a few with contra indications**	5 (2)
Without statistical analysis	
Previous literature	5 (2)
Previous literature and few variables by investigator choice	5 (2)
By statistical analysis	
Screening by univariable analysis - only significant variables	37 (16)
Screening by univariable analysis - significant variables and investigator choice	11 (5)
Unclear/Not reported	11 (5)

**Statistical modelling methods used within multivariable analysis**	
A priori variables fixed, others added	2 (1)
Backward elimination	14 (6)
Forward selection	5 (2)
Other (pairwise multiple testing for categories of variables)	2 (1)
Unclear/Not reported	77 (33)

**Methods for inclusion of variables in final model and prognostic index**	
No selection. All variables kept in model	14 (6)
Retain only significant variables based on P-value	65 (28)
Retain significant variables plus variables based on previous literature	2 (1)
Retain all variables but alter prognostic score after model to include only significant variables and adjust for other variables	5 (2)
Retain only significant variables but alter prognostic score after final model	5 (2)
Retain based on model *goodness of fit*	2 (1)
Unclear/Not reported	7 (3)

* Excluded four studies using recursive partitioning analysis and artificial neural network models

** Contra indications reported as reasons for exclusion of variables were missing data, collinearity and treatment indicator

The statistical modelling methods used within the multivariable models were reported in only 33% (10) of the studies (Table 4). In one model the selection method started with fixed *a priori *variables and added further variables [26], a method likely to be appropriate where there is much established clinical knowledge. Backward elimination was used in 14% (6) of the models and the less recommended forward selection in 5% (2).

Fourteen percent (6) of the prognostic models were based on a multivariable analysis in which all candidate variables were included regardless of their statistical significance. We note that variables will almost certainly have been selected from those clinically recorded, so it is unlikely variables are in reality completely unselected [5]. In 65% (28) of the models researchers retained only variables statistically significant in multivariable modelling, with one model also retaining variables established as important in the previous literature [38]. One model was based on variables selected using *goodness of fit *of the model as a whole [51], an alternative method to selecting by P-value of individual variables [3].

In two models researchers retained only significant variables in the final model, but then added to or removed variables from the prognostic score without repeating the model fit, based on investigators' choice [29,58]. The authors of two articles appear confused as to the purpose of their models, and included adjustment for six to nine variables in a footnote to their model, while retaining only two significant variables in the predictive score [18,43]. Effectively the adjusted variables are altering the coefficient values of the variables in the score, but are not available to readers. In 7% (3) of the models authors did not report methods used to include variables in the final model [44,55,57].

Overall 87% (37) of models were developed using selection of variables during modelling (Table 3), from a median of 11 candidate variables (IQR 7 to 14; range 2 to 27; n = 46) used to develop models to a median of four variables included (IQR 3 to 6, range 2 to 12, n = 44) in the final multivariable model (Table 3). Calculation of the number of final variables in the model excluded four studies, two studies where the final model was not reported and two where the final model was not clear.

### Examples of good methods and reporting

Although the quality of the articles was generally disappointing, we particularly wish to highlight two articles using good methods and good reporting [38,40]. These two studies deserve mentioning as authors included: good study design for developing prognostic models e.g. RCTs; sufficient sample sizes to allow reliable model development (EPV values of over 40); the coding of variables was clear and not all continuous variables categorised; reporting of the model method and coefficients for the final model; reasonable strategies for selection of variables were used; and proportional hazards assumptions tested for the Cox model. The reporting of the definition of deaths included in overall survival could have been clarified. Also, it could have been noted whether the use of complete cases in multivariable analysis was likely to have introduced selection bias in these RCT patient populations. These articles [38,40] in combination with published guidelines on development of prognostic models [1-5,61] may be useful to researchers developing prognostic models.

## Discussion

In this article we have highlighted the methods currently used to develop prognostic models for clinical predictions about patients with cancer, and the poor reporting of those methods. The quality of prognostic models depends on study design and statistical methods. Researchers should understand the assumptions inherent in the statistical methods and follow sound principles to ensure that methods are appropriately applied and reported [75].

Most prognostic models, 68% in this study, are based on retrospective databases which have many inherent biases, often due to patchy availability of patient information [60]. The lack of studies specifically designed to meet patients' and healthcare professionals' needs for prognostic information is highlighted by the fact that none of the studies in our sample justified the chosen sample size.

The paucity of prognostic models based on prospective cohorts is a serious limitation in the generalisability and usefulness of quantitative information valued by both patients and healthcare professionals. Randomised controlled trials could potentially provide good datasets for model development if they include sufficiently detailed baseline data and coverage of the relevant patient population.

Fifty percent (14 of 28) of the studies, where EPV could be calculated, had fewer than 10 events per candidate variable (EPV) used in model development, with only eight studies having over 20 events per variable. In at least half of these models it is probable that many of the variables selected as statistically significant would not be selected if the modelling was repeated in a similar sample, due to the low number of events in the sample and the investigator choice of variables and methods [5,63]. It follows that many of these models are unreliable. In any case, all newly proposed models need independent validation before being considered for clinical use [76]. Other reviewers of prognostic studies have also found a large number of both Cox and logistic models with <10 EPV [65,77].

Poor reporting was evident in all aspects of model development, from descriptions of the patient data to statistical modelling methods, including details of the multiple steps where bias or errors can be introduced (Table 4). In only 60% (28) of the studies did authors include clear reporting of outcome events used in the model (Table 2). Lack of transparency in the definition of outcomes has been shown to lead to lack of transparency and transportability in studies [67].

Coding of variables used in model development and methods used to select variables in the multivariable models are also critical to the reliability of modelling methods. Continuous variables should be retained in the model as continuous measures, as otherwise information is lost and results can be biased by choice of cutpoints used in variable categorisation. Fractional polynomial functions [5] and splines are recommended methods for modelling continuous variables that have nonlinear relationships with the outcome variable. In only one article [13] were all candidate continuous variables kept as continuous in developing the model (Table 3). Categorisation was applied at least to some continuous variables in 70% of models. In about a third of the models it was unclear about how continuous variables were coded. Poor reporting of coding of variables in both Cox and logistic models has also been reported in other studies [65,78,79].

There was evidence that poor methods are widely used for the selection of variables for models, as a method that is not recommended was used in 48% of studies. This method was pre-screening of variables for model inclusion based on univariable analysis [4]. Univariable screening can lead to rejection of variables that could have been influential prognostic factors [74], especially with small sample sizes and in the presence of collinearity between variables. Poor reporting of variable selection has been described similarly in other studies [65,80].

Although in this study we limited attention to prognostic models in cancer, the problems identified are not specific to cancer. These models had a time to event outcome, and Cox regression modelling was used in almost all studies. However, many issues identified here are similar to those found in logistic regression.

This research, together with the companion article Mallett et al [8], highlights poor methods and reporting in the development of prognostic models in published articles. There is a need for researchers to use more appropriate methods and to report their studies more effectively. In addition, peer reviewers and journal editors need to be more demanding in their assessment of articles for publication.

## Conclusions

In conclusion we found that published prognostic models are often characterised by both use of poor methods for development of multivariable models and poor reporting. In addition, models are limited by the lack of studies based on prospective data of sufficient sample size to avoid overfitting.

Prognostic models are developed to provide objective probability estimates to complement clinical intuition of the physician and guidelines [81]. Many published prognostic models have been developed using poor methodological choices that may adversely affect model reliability.

## Abbreviations

DFS: disease free survival; EPV: events per variable; IQR: interquartile range; MeSH: medical subject heading from the U.S. National Library of Medicine's controlled vocabulary; NLM: National Library of Medicine; RCT: randomised controlled trial.

## Competing interests

The authors declare that they have no competing interests.

## Authors' contributions

SM contributed to the design, carried out data extraction on all articles and items, compiled results and drafted the manuscript. PR and DGA contributed to the design and drafting of the article. RW and SD carried out duplicate data extraction for some data items and commented on the manuscript.

## Authors' informations

All authors are medical statisticians.

## Pre-publication history

The pre-publication history for this paper can be accessed here:

http://www.biomedcentral.com/1741-7015/8/20/prepub

## Supplementary Material

Additional file 1 **Search string Mallett 2009**. This file includes the search strategy and Pubmed search string format. Click here for file

Additional file 2 **Comparison of prognostic search strings**. Part (A) includes the performance of hand search and search string. Part (B) is a comparison of the included articles found with other prognostic search strings. Click here for file
