# Supplementary material for: Reporting methods in studies developing prognostic models in cancer: a review
Source: BMC Med. 2010 Mar 30;8:20. doi: 10.1186/1741-7015-8-20 (PMC2856521; doi:10.1186/1741-7015-8-20)
Supplement: Additional file 1 — Search string Mallett 2009. This file includes the search strategy and Pubmed search string format. [file 1741-7015-8-20-S1.DOC]

**Additional file 1: Search string Mallett 2009**

Search date: 231006

Number of hits: 2076

Search software: PubMed from U.S. National Library of Medicine

Search term syntax: PubMed

| **Step** | **Search strategy** | **Pubmed search string format** |
| --- | --- | --- |
| #1  AND | Cochrane Collaboration cancer search string | (neoplasms[All Fields] OR neoplas*[All Fields] OR (tumour[All Fields] OR tumor[All Fields]) OR melanoma[All Fields] OR cancer[All Fields] OR malignan*[All Fields] OR (leukemia[All Fields] OR leukaemia[All Fields]) OR carcin*[All Fields] OR metastas*[All Fields] OR sarcoma[All Fields]) |
| #2 AND | Pubmed filter for articles indexed under disease category "neoplasms" | neoplasms[MeSH Terms] |
| #3  AND | Restriction to articles published in 2005 | 2005[Publication Date] |
| #4  NOT | Excluding study designs not included in study | (expression profiling[All Fields] OR microarray*[All Fields] OR proteomic*[All Fields] OR affymetrix[All Fields]) |
| #5  AND | Search terms for prognostic studies identified from handsearches | (prognostic scor* OR prognostic index OR nomogram OR predictive model OR validation OR validate OR prognostic model OR predictor) AND (scor* OR index OR model OR predict* OR nomogram OR validat*) |
| #  NOT | Excluding publication types that are not appropriate to study | (review[Publication Type] OR Bibliography[Publication Type] OR Editorial[Publication Type] OR Letter[Publication Type] OR Meta-analysis[Publication Type] OR News[Publication Type]) |
