# Supplementary material for: Reporting methods in studies developing prognostic models in cancer: a review
Source: BMC Med. 2010 Mar 30;8:20. doi: 10.1186/1741-7015-8-20 (PMC2856521; doi:10.1186/1741-7015-8-20)
Supplement: Additional file 2 — Comparison of prognostic search strings. Part (A) includes the performance of hand search and search string. Part (B) is a comparison of the included articles found with other prognostic search strings. [file 1741-7015-8-20-S2.DOC]

**Appendix 2: Comparison of prognostic search strings**

(A) Performance of hand search and search string

| **Search string** | **Journals** | **Total articles** | **Title & summary** | **Title & abstract** | **Full paper** | **Included** |
| --- | --- | --- | --- | --- | --- | --- |
| Hand search | Cancer | 784 | 784 | 42 | 16 | 4 |
| Mallett 2009 | Cancer | 784 | NA | 74 | 12 | 5 |
| Mallett 2009 | all pubmed  cancer  not reviews | 681,530 | NA | 2076 | 99 | 47 |

(B) Comparison of included articles found with other prognostic search strings

| **Prognostic search string used in conjunction with subject and article type strings** | **Number hits from**  **search string** | **Percentage included articles found (n=47)** | **Percentage included articles of total hits** |
| --- | --- | --- | --- |
| Mallett 2009 | 2076 | 100% (47) | 2.3% |
| Ingui 2001 search string ref #T2/F2 | 8610 | 91% (43) | 0.5% |
| Wilczynski 2004  Clinical prediction narrow and cancer 2005 | 786 | 15% (7) | 0.9% |
| Wilczynski 2004  Prognosis narrow and cancer 2005 | 9411 | 89% (42) | 0.4% |
